# Self-Sustained Oscillation of Electrothermally Responsive Liquid Crystal Elastomer Film in Steady-State Circuits

**DOI:** 10.3390/polym15132814

**Published:** 2023-06-25

**Authors:** Junxiu Liu, Zongsong Yuan, Junjie Zhao, Yuntong Dai, Kai Li

**Affiliations:** 1 Anhui Province Key Laboratory of Building Structure and Underground Engineering, Anhui Jianzhu University, Hefei 230601, China; tjuliu@ahjzu.edu.cn; 2 College of Civil Engineering, Anhui Jianzhu University, Hefei 230601, China; ys2523578175@163.com (Z.Y.); zhaojunjie0913@163.com (J.Z.); daiytmechanics@ahjzu.edu.cn (Y.D.)

**Keywords:** Self-excited motion, liquid crystal elastomers, Self-oscillation, electrothermally responsive, dynamic boundary problem

## Abstract

Self-excited oscillations have the advantages of absorbing energy from a stable environment and Self-control; therefore, Self-excited motion patterns have broader applications in micro devices, autonomous robots, sensors and energy-generating devices. In this paper, a Self-sustained curling liquid crystal elastomer (LCE) film-mass system is proposed on the basis of electrothermally responsive materials, which can realize Self-oscillation under a steady-state current. Based on the contact model and dynamic LCE model, a nonlinear dynamics model of LCE film in steady-state circuits is developed and numerical calculations are carried out using the Runge–Kutta method. Through numerical calculations, it is demonstrated that LCE film-mass systems have two motion patterns in steady-state circuits: namely, a Self-oscillation pattern and a stationary pattern. Self-sustained curling of LCE film originates from the fact that the energy absorbed by the system exceeds the energy dissipated due to the damping effect. In addition, the critical conditions for triggering Self-oscillation and the effects of several key dimensionless system parameters on the amplitude and period of Self-oscillation are investigated in detail. Calculation results show that the height of electrolyte solution, gravitational acceleration, elastic modulus of LCE film, limit temperature, curvature coefficient, thermal shrinkage coefficient and damping factor all have a modulating effect on the amplitude and period of Self-oscillation. This research may deepen the understanding of Self-excited oscillation, with promising applications in energy harvesting, power generation, monitoring, soft robotics, medical devices, and micro and nano devices.

## 1. Introduction

Self-excited oscillation is a phenomenon in which a system generates periodic motion in response to a constant external stimulus [1,2,3,4,5]. Firstly, Self-excited oscillation largely reduces the requirement for a system controller, since its periodic motion requires a constant external stimulus rather than a periodic stimulus [6,7]. Secondly, the nature of Self-excited oscillation determines its active energy absorption from an external stable environment to maintain its own periodic motion. Furthermore, the amplitude and period of Self-excited oscillations usually depend on the intrinsic properties of the system, which contribute to the high robustness of the system [8]. Finally, based on the above-mentioned passive and control-free features, they can reduce the system complexity for intelligence and automation, as well as save resources and increase efficiency [9,10]. This allows for a wide range of potential applications in energy control [11,12], autonomous robotics [13,14,15], micro–nano devices [16] and medical devices [17,18,19].

A rich variety of Self-excited oscillation systems constructed based on active materials have been recently reported, such as hydrogels [20,21], dielectric elastomers, ionic gels [22,23], liquid crystal elastomers (LCEs) [24,25,26,27,28,29] and temperature-sensitive polymers [30,31,32,33]. At the same time, researchers have proposed and constructed different Self-excited oscillation patterns based on various types of active materials, such as bending [30,31,32], flexing, twisting [33,34], stretching and contracting [35], rolling [36], swimming, oscillating, vibrating [37,38,39], jumping [40,41,42], rotating [43], turning outward or reversing, and even the synchronized motion of several coupled Self-oscillators. Owing to the damping effect of systems, Self-excited oscillation consumes energy during motion, so it usually originates from nonlinear feedback mechanisms that compensate for the damping consumption of a system through energy input [44,45,46], such as the Self-shadowing mechanism [36,47], the coupling mechanism of chemical reaction and large deformation [22], and the coupling motion mechanism of air expansion and liquid column [48,49].

LCEs are important electrothermally responsive materials consisting of rod-like mesocrystalline monomers with flexible cross-linked polymer backbones or side chains that combine the elasticity of rubber with the anisotropy of liquid crystals. When liquid crystal monomer molecules receive external stimuli such as electricity, heat, light and magnetism, they will undergo rotations or phase transitions to change their conformation, causing macroscopic deformation [50,51,52,53,54,55]. Compared with other types of active materials such as temperature-sensitive gels, moisture-sensitive gels, pneumatic artificial muscles and polyelectrolyte gels, in addition to general advantages of high sensitivity, reversibility and repeatability, they also have unique advantages such as high controllability, high stability and wireless non-contact actuation. Self-excited oscillation systems based on LCE materials have received much attention, and these Self-excited oscillation systems have a wide range of potential applications in energy control [56,57], autonomous robotics [58,59], micro and nano devices [16] and medical devices.

Those Self-excited oscillation systems [60,61] based on LCE materials that have been built are usually driven by direct ambient heating or by photothermal and photochemical effects [29,48,62,63,64,65,66,67,68,69]. However, the current variety of Self-excited oscillation patterns is not yet sufficient, which limits the application of Self-excited oscillation phenomena in active motors. For most practical applications, electronically powered actuators offer notable convenience for system control and integration. Several recent studies have successfully integrated stretchable resistive heaters into LCEs [70,71,72], whose actuation can then be easily controlled by an electrical potential. In this paper, a Self-sustained curling LCE film-mass system is proposed. An LCE film carrying mass blocks at both ends is placed on an experimental bench to achieve Self-sustained curling in a steady-state circuit. During this Self-sustained motion, there is a complex dynamic boundary problem as the contact range between the bottom of the system and the table changes continuously.

The rest of the current paper is organized as follows: in Section 2, the governing equations for the dynamics of LCE film-mass systems in steady-state circuits are established based on the dynamic LCE model. The Self-oscillation motion of the LCE film-mass system is calculated numerically in Section 3 using the Runge–Kutta method. The two motion patterns of the LCE film-mass system in steady-state circuits, i.e., the Self-oscillation pattern and the stationary pattern, are investigated, and the mechanism of Self-oscillation is explained in detail. Section 4 performs a parametric analysis to investigate the effect of each parameter on the onset condition, proximity condition, amplitude and period of the Self-oscillation motion. Finally, the conclusion is given.

## 2. Model and Formulation

In this section, a Self-sustained curling LCE film-mass system in a steady-state circuit is constructed, and a theoretical model of a Self-curling LCE film considering dynamic boundary conditions is developed based on the contact model and the dynamic LCE model. The main content includes the dynamics of the Self-oscillating LCE film, the solution method for the differential control equations with variable coefficients, and the dimensionless process of the system parameters.

### 2.1. Dynamics of a LCE Film-Mass System

**Figure 1:**
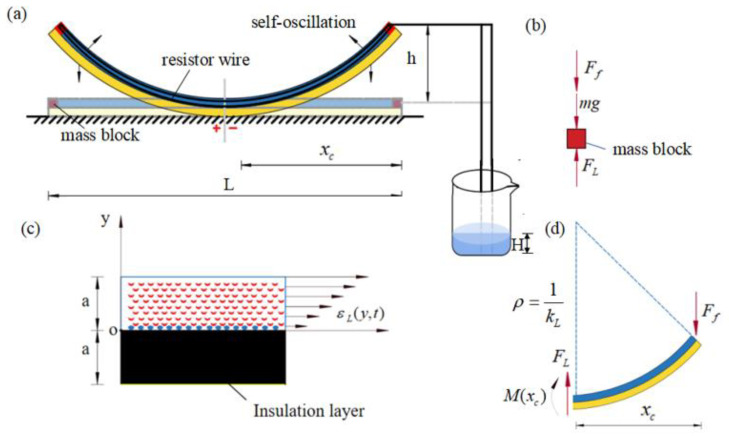
(**a**) Schematic diagram for the dynamics of a Self-sustained curling LCE film-mass system in a steady-state circuit. (**b**) Force analysis for the mass block at the end of the LCE film, which is subjected to mass gravity mg, damping force $F_{f}$ and elastic force $F_{L}$ provided by the LCE film. (**c**) Enlarged cross-sectional view of the LCE film, showing the electrothermally driven strain distribution in the LCE film, where the upper layer is the LCE film and the lower layer is the insulation layer. (**d**) Force analysis for the non-contact part of the system with the table, which is subjected to elastic force $F_{L}$, cross-sectional shear force and bending moment provided by the contact part. In steady-state circuits, the LCE film can curl periodically in a Self-oscillation motion.

Figure 1 presents the schematic diagram of an electrothermally responsive LCE film-mass system that can maintain Self-sustained curling in steady-state circuits. As shown in Figure 1a, the overall system consisting of an LCE film with internal resistance wire and an insulation layer is placed on a horizontal table, and two mass blocks of mass m are attached to both ends of the LCE film. The length of the LCE film is L, and the thickness is a. It is assumed that the mass of the LCE film as well as the resistance wire is much smaller than the mass of the mass block, thus meaning that the inertia of the LCE film is neglected. A piece of wire connected to the internal resistance wire is led from the side of the LCE film, one end of the wire is placed at the bottom of the beaker and a height of electrolyte solution H is poured into the beaker. At this point, the circuit is connected into a pathway; only the resistance wire inside the LCE film is energized, generating heat due to the thermal benefit of the current. Due to uneven electrothermally driven contraction along the thickness direction, the LCE film will bend upwards, thus pushing the mass block upwards. When the mass rises above H, the end of the wire leaves the electrolyte solution and the circuit is broken. Subsequently, the LCE film bends backwards due to the recovery of electrothermally driven contraction as the temperature drops, prompting the mass block to move rapidly downwards. As the mass block moves rapidly downwards, the end of the wire enters the electrolyte solution again and the circuit reverts to a path, causing the mass block to subsequently be driven upwards again. Ultimately, the LCE film-mass system allows for Self-sustained curling under a steady-state circuit. It is worth noting that the dimensions of the contact surface between the LCE film and the horizontal table are continuously changing during Self-sustained curling of the LCE film, thus creating a dynamic boundary problem.

To describe the current state of the LCE film-mass system, we introduce a y-axis in the vertical direction. The position of the mass block at moment *t* is denoted as h(t), and given the symmetry of this problem, we choose one half of the LCE film-mass system for analysis, as shown in Figure 1b. We emphasize that the middle symmetry plane of the LCE film does not rotate, which corresponds to a fixed end constraining the bending of the LCE film. The LCE film acts an elastic force $F_{L}$ on the mass block at its end, which also receives mass gravity mg and damping force $F_{f}$. Since the eigenperiod of the LCE film can be adjusted by varying the mass of the mass block and the bending stiffness of the LCE film, for comparable eigenperiods and characteristic times of thermal response characteristic times, the kinetic equation controlling the motion of the mass block can be derived from the force equilibrium as [73],

(1) $$m\ddot{h}(t)=F_{L}(t)-mg-F_{f}(t)$$

where g is gravitational acceleration and $\ddot{h}$ denotes the acceleration of the mass block. For simplicity, it is assumed that the damping force $F_{f}$ is proportional to the velocity v, namely

(2) $$F_{f}=\beta v=\beta \dot{h}(t)$$

where β is the damping factor and $\dot{h}(t)$ is the velocity of the mass block.

In order to calculate the elastic force of the LCE film acting on the mass block, the electrothermally driven curvature $k_{L}$ of the LCE film needs to be determined first. Given that the film is uniformly distributed inside due to the resistance wire, the temperature variation of the LCE film is assumed to be uniform for the simplicity of modeling. For LCE film in the energized state, the uniform electrothermally driven strain varying along the thickness direction is denoted as ε(y,t)L. According to [73], the uniform electrothermally driven strain leads to electrothermally driven curvature of the LCE film, namely

(3) kL(t)=∫0aε(y,t)LydyIZ

where $I_{Z}$ is the principal moment of inertia for the total cross-section of the LCE layer and the insulation layer.

In general, at any instant, the electrothermally driven curvature of the LCE film is $k_{L}$, the current position of the mass block is h(t), and the LCE film is in partial contact with the table. The length of the LCE film leaving the table is $x_{c}(t)$, which varies continuously with the Self-sustained curling of the LCE film, and is yet to be quantified. The curvature at contact is zero, so the bending moment $M(x_{c})$ can be expressed as

(4) $$M(x_{c})=EI_{Z}(k_{L}-0)$$

where E is the total elastic modulus of the LCE layer and the insulation layer.

At the same time, we need to consider the equilibrium of the non-contact part of the system with the table (Figure 1d), namely

(5) $$M(x_{c})=F_{L}x_{c}(t)$$

Combining Equations (4) and (5), the non-contact length $x_{c}(t)$ of the system with the table is expressed as

(6) $$x_{c}(t)=\frac{EI_{Z}k_{L}(t)}{F_{L}(t)}$$

As shown in Figure 1d, the deflection of the beam end can be expressed as w=h−h(t), and is also related to the elastic force $F_{L}$ as $w=\frac{F_{L}X_{c}^{3}(t)}{3EI_{Z}}$ according to classical beam theory. Therefore, we have

(7) $$h-h(t)=\frac{F_{L}X_{c}^{3}(t)}{3EI_{Z}}$$

where the initial position of the beam end $h=\frac{1}{k}-\sqrt{\frac{1}{k^{2}}-x_{c}^{2}}$.

Combining Equations (6) and (7), the analytical formula of the elastic force $F_{L}$ is

(8) $$F_{L}=\pm \frac{\sqrt{2}EI_{Z}k_{L}^{2}(t)}{-3-6k_{L}(t)h(t)+3\sqrt{12k_{L}(t)h(t)-4k_{L}^{2}(t)h^{2}(t)+1}}$$

Substituting Equations (2) and (8) into Equation (1), the dynamic equation for the mass block is obtained as

(9) $$m\ddot{h}(t)=\frac{\sqrt{2}EI_{Z}k_{L}^{2}(t)}{-3-6k_{L}(t)h(t)+3\sqrt{12k_{L}(t)h(t)-4k_{L}^{2}(t)h^{2}(t)+1}}-mg-\beta \dot{h}(t)$$

It is worth noting that Equation (9) is determined by Equation (3). For $F_{L}=0$ in Equation (9), the system remains stationary because there is zero current, and for $F_{f}=0$, the amplitude of Self-oscillation may approach infinity due to no damping, which is beyond the scope of this study. Temperature changes can drive deformation of the electrothermally responsive LCE film, assuming that thermal strain in the LCE film is linearly related to temperature changes. In addition, the elastic modulus is assumed to be large and elastic strain is neglected. Therefore, the length of the LCE film can be calculated as

(10) $$\varepsilon_{L}=\varepsilon_{0}(1-\alpha T)$$

where $\varepsilon_{0}$ is the starting length of the LCE film without thermal strain, α is the linear thermal shrinkage coefficient of the LCE, and T is the temperature difference between the LCE film and the environment.

### 2.2. Temperature Field in LCE

This section describes the dynamics of the temperature and length of the LCE film under energized and de-energized conditions. It is assumed that heat exchange within the LCE film is very fast and the temperature within the LCE film is uniform. Due to the current thermal effect, the resistance wire inside the LCE film will generate heat when energized. Heat generated per second by the thermal effect of the current is denoted by q. The LCE film can also exchange heat with the environment, assuming that the heat flow density is linearly related to the temperature difference T between the LCE layer and the environment.

In the case of the energized condition, the temperature difference T is [43]

(11) $$\dot{T}=\frac{q-KT}{\rho_{c}}$$

(12) $$T=T_{L}-T_{e}$$

where $\rho_{c}$ is the specific heat capacity, K is the heat transfer coefficient, $T_{L}$ is the temperature of the LCE film and $T_{e}$ is the ambient temperature. 

In the case of the energized condition, the temperature difference T is

(13) $$T_{n+1}=T_{n}+\frac{t}{\tau }(T_{0}-T_{n})$$

where $T_{0}=q/K$ represents the limit temperature difference of the electrothermally responsive LCE film under a long-time energized condition; $\tau =\rho_{c}/K$ reflects the characteristic time for heat exchange between the LCE film and the environment. A larger τ indicates a longer time required to attain the limit temperature difference $T_{0}$ of the LCE film.

In the case of the de-energized condition, when $T_{0}=0$, the temperature difference T is

(14) $$T_{n+1}=T_{n}-\frac{t}{\tau }T_{n}$$

Combining Equations (3), (10) and (11) yields that the curvature $k_{L}(t)$ formed at the end mass during Self-oscillation is proportional to the thermal strain $\varepsilon_{L}$ of the LCE film as

(15) kL(t)=∫0aε(t)LydyIZ=a22IZε(t)L

(16) kL(t)=Aε(t)L

where A represents the curvature coefficient, namely $A=\frac{a^{2}}{2I_{Z}}$.

### 2.3. Nondimensionalization and Solution

For convenience, the dimensionless quantities are introduced as follows: $\bar{t}=\frac{t}{\tau }$, ${\bar{I}}_{Z}=\frac{I_{Z}}{L^{4}}$, $\bar{\beta }=\frac{\beta \tau }{m}$, $\bar{H}=\frac{H}{L}$, $\bar{g}=\frac{g\tau }{L}$, $\bar{h}=\frac{h}{L}$, $\bar{E}=\frac{EI_{Z}\tau^{2}}{mL^{3}}$, ${\bar{k}}_{L}=k_{L}L$, $\bar{A}=\frac{a^{2}L^{2}}{2I_{Z}}$, $\bar{T}=\frac{T}{T_{e}}$, ${\bar{T}}_{0}=\frac{T_{0}}{T_{e}}$ and $\bar{\alpha }=\alpha T_{e}$. From Equation (8), the dimensionless elastic force can be

(17) $${\bar{F}}_{L}=\frac{\sqrt{2}\bar{E}{\bar{k}}_{L}^{2}}{\sqrt{-3-6{\bar{k}}_{L}\bar{h}+3\sqrt{12{\bar{k}}_{L}\bar{h}-4{\bar{k}}_{L}^{2}{\bar{h}}^{2}+1}}}$$

The governing Equation (9) can be nondimensionalized with the following

(18) $$\bar{\ddot{h}}=\frac{\sqrt{2}\bar{E}{\bar{k}}_{L}^{2}}{\sqrt{-3-6{\bar{k}}_{L}\bar{h}+3\sqrt{12{\bar{k}}_{L}\bar{h}-4{\bar{k}}_{L}^{2}{\bar{h}}^{2}+1}}}-\bar{g}-\bar{\beta }\bar{h}$$

From Equation (16), the dimensionless curvature can be expressed as

(19) k¯L(t¯)=A¯ε¯(t¯)L

The initial conditions are that when $\bar{t}=0$,

(20) $$\bar{h}={\bar{h}}_{0},\bar{\dot{h}}={\bar{\dot{h}}}_{0}$$

Taking into account the dimensionless parameters including $\bar{H}$, $\bar{g}$, $\bar{E}$, ${\bar{T}}_{0}$, $\bar{A}$, $\bar{\alpha }$ and $\bar{\beta }$, Equations (17)–(20) govern the motion of LCE film-mass systems in steady-state circuits. To solve the complex differential Equation (18) with variable and process-related coefficients, we perform numerical calculations in the software Matlab based on the well-known fourth-order Runge–Kutta method. Through carrying out a convergence analysis, the time step *h* = 0.001 was set. In the calculation, we give the LCE film an initial displacement. For the previous position ${\bar{h}}_{\left(i-1\right)}$ and the previous curvature ${\bar{k}}_{L(i-1)}$, we can calculate the corresponding elastic force ${\bar{F}}_{L(i-1)}$ according to Equation (17). The current position ${\bar{h}}_{i}$ can be further calculated from Equation (18), and the current curvature ${\bar{k}}_{i}$ can be calculated from Equation (19). When the current position ${\bar{h}}_{{}_{i}}<\bar{H}$, the end of the wire is in the electrolyte solution and the circuit is energized; when the current position ${\bar{h}}_{{}_{i}}>\bar{H}$, the end of the wire is above the electrolyte solution and the circuit is de-energized. According to the current curvature ${\bar{k}}_{i}$, we can obtain the current elastic force ${\bar{F}}_{i}$ by Equation (17). Then we proceed to obtain position ${\bar{h}}_{\left(i+1\right)}$ and curvature ${\bar{k}}_{L(i+1)}$ of the LCE film in turn from Equations (18) and (19). By iterative calculations, the time history of the position of the LCE film-mass system can be obtained and the effects of different parameters on its Self-oscillation can be further investigated.

## 3. Two motion Patterns and Mechanism of Self-Oscillation

Considering the above control equations, the dynamic behavior of Self-sustained curling LCE film-mass systems in steady-state circuits is investigated by numerical calculations. We first present two motion patterns, namely a stationary pattern and a Self-oscillation pattern. Then the corresponding mechanism of Self-oscillation is elaborated through parametric analysis.

### 3.1. Two Motion Patterns

In order to study Self-sustained curling of LCE film-mass systems, it is first necessary to determine the dimensionless parameters in the theoretical model. Taking data from existing experiments [74,75], the material properties and geometric parameters of the system, as well as the corresponding dimensionless parameters, are listed in Table 1 and Table 2, respectively. In this paper, the following parameter values are used to study the Self-oscillation of LCE film-mass systems in steady-state circuits.

**Figure 2:**
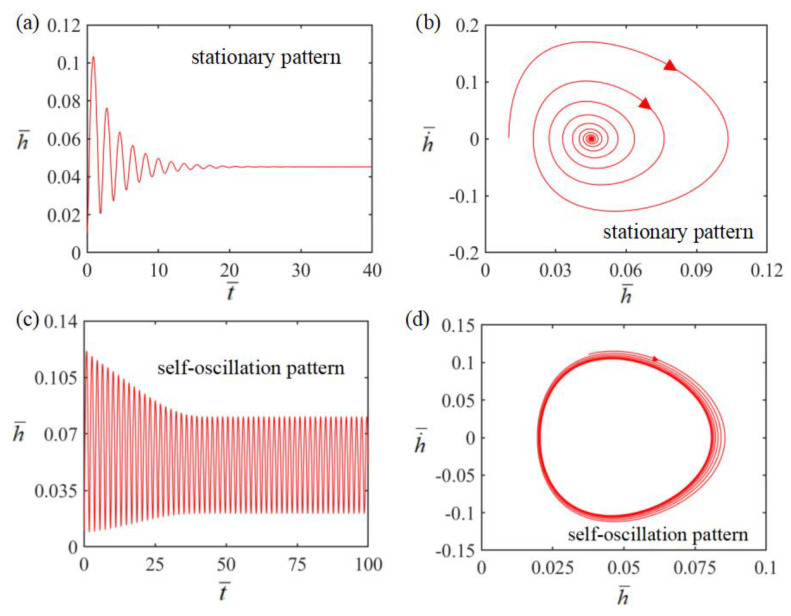
(**a**,**b**) Plots showing the time course and phase trajectory plots of the steady-state orientation diagram of the LCE membrane-mass system under the conditions of parameters $\bar{H}=0.08$, $\bar{g}=1.2$, $\bar{E}=2.5$, $\bar{A}=0.40$, $\bar{\alpha }=0.35$, $\bar{\dot{h}}=0$, $\bar{\beta }=0.001$ and ${\bar{T}}_{0}=0.05$. (**c**,**d**) Time course and phase trajectory plots of the Self-oscillation pattern of the LCE thin film-mass system under the conditions of parameters $\bar{H}=0.08$, $\bar{g}=1.2$, $\bar{E}=2.5$, $\bar{A}=0.40$, $\bar{\alpha }=0.35$, $\bar{\dot{h}}=0$, $\bar{\beta }=0.001$ and ${\bar{T}}_{0}=0.1$. There are two modes of motion of the LCE thin film-mass system under steady-state circuits: fixed pattern and Self-oscillation pattern.

Next, the Self-oscillation phenomenon of LCE film in steady-state circuits will be investigated using these key parameters. From Equations (17) and (18), the time history and phase trajectory for the Self-oscillation motion of LCE film can be obtained. In the calculation, we first set $\bar{H}=0.08$, $\bar{g}=1.2$, $\bar{E}=2.5$, $\bar{A}=0.40$, $\bar{\alpha }=0.35$, $\bar{\dot{h}}=0$, $\bar{\beta }=0.001$ and ${\bar{T}}_{0}=0.05$ At this time, the end of the wire is located in the electrolyte solution and the system is in a steady-state circuit; the temperature rises due to the thermal effect of the current, and the LCE film shrinks and begins to bend upwards in response to the thermal drive. When the end of the wire leaves the electrolyte solution, the circuit is broken and the temperature starts to drop due to heat transfer, and the LCE film is promoted to bend downwards due to the recovery of electrothermally driven contraction. During this motion, the oscillation amplitude of the system decreases continuously due to air damping and finally remains stable, which is called the stationary pattern, as shown in Figure 2a,b. Next we set $\bar{H}=0.08$, $\bar{g}=1.2$, $\bar{E}=2.5$, $\bar{A}=0.40$, $\bar{\alpha }=0.35$, $\bar{\dot{h}}=0$, $\bar{\beta }=0.001$ and ${\bar{T}}_{0}=0.1$. The oscillation amplitude of the system first decreases and then remains constant, as shown in Figure 2c,d. This result implies that the LCE film can be continuously bent in a steady-state circuit, eventually presenting a Self-sustained curling motion, called the Self-oscillation pattern. Like other Self-oscillating systems, the LCE film can perform Self-sustained curling in a steady-state circuit, mainly because the energy input from electrothermal conversion compensates for damping dissipation, thus maintaining Self-curling. In Section 3.2, we will explore the mechanism of Self-sustained curling in detail.

### 3.2. Mechanism of Self-Oscillation

**Figure 3:**
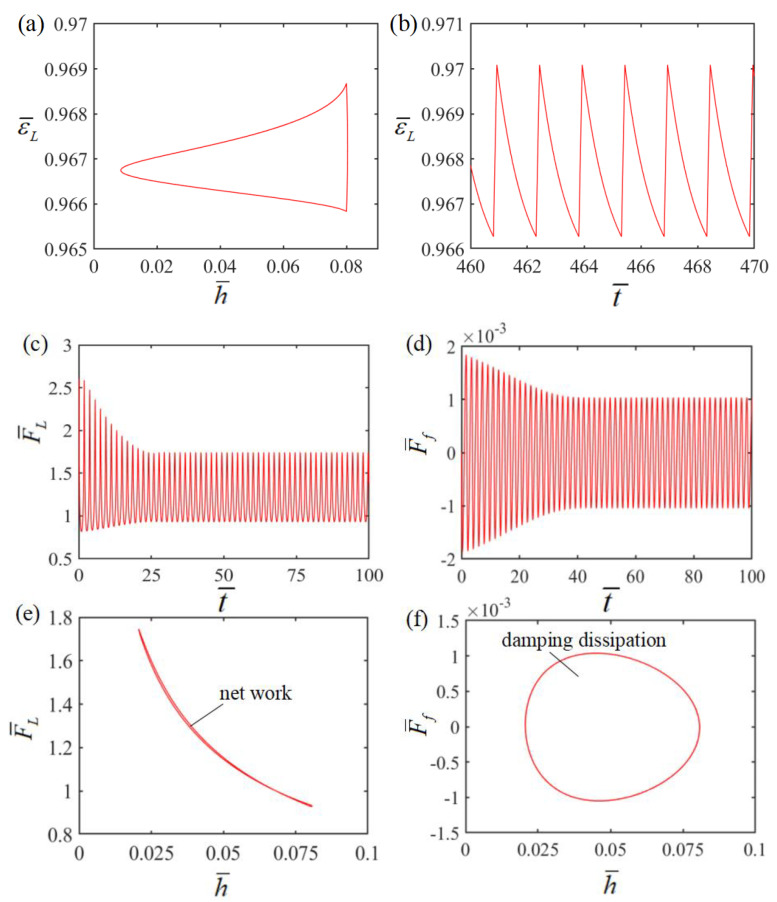
(**a**) Time dependence of electrothermally driven strain in LCE film; (**b**) displacement dependence of electrothermally driven strain in LCE film; (**c**) time dependence of the elastic force; (**d**) time dependence of the damping force; (**e**) displacement dependence of the elastic force; (**f**) displacement dependence of the damping force.

**Figure 4:**
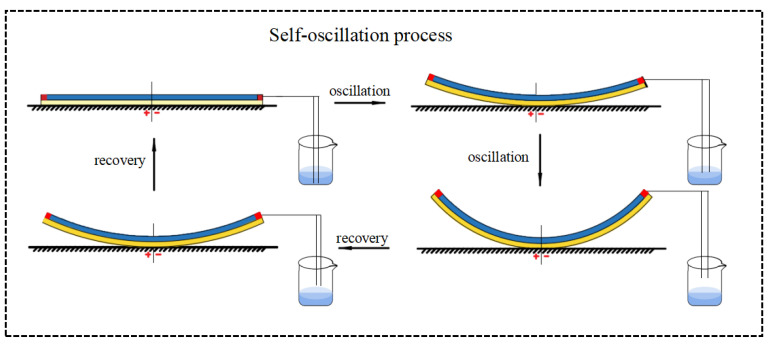
Figure 4 shows the Self-excited oscillation of an LCE film-mass system in a steady-state circuit.

In order to investigate the mechanism of Self-oscillation, Figure 3 shows the evolutions for several key parameters of Self-sustained curling in Figure 2c,d. Figure 4 shows Self-excited oscillation of an LCE film-mass system in a steady-state circuit. Figure 3a,b plots the time dependence and displacement dependence of length change ${\bar{\varepsilon }}_{L}$ in LCE material due to electrothermally driven shrinkage. The time dependence of the elastic force ${\bar{F}}_{L}$ is plotted in Figure 3c, showing a periodic variation with time. Figure 3d plots the time dependence of damping force ${\bar{F}}_{f}$, also showing a periodic variation with time. The displacement dependence of the elastic force is plotted in Figure 3e. A closed curve is formed in one cycle, with the enclosed area representing the net work done by the elastic force, which is calculated as 4.56 × 10^−5^. Meanwhile, the displacement dependence of the damping force is shown in Figure 3f. A closed curve is also formed in one cycle, with the enclosed area representing the damping dissipation energy, which is calculated as 4.56 × 10^−5^. The positive net work done by elastic force ${\bar{F}}_{L}$ exactly compensates for the damping dissipation generated by the damping force ${\bar{F}}_{f}$, and thus continuous Self-sustained curling can be maintained.

## 4. Parametric Study

Seven dimensionless parameters appear in the above equations: $\bar{H}$, $\bar{g}$, $\bar{E}$, ${\bar{T}}_{0}$, $\bar{A}$, $\bar{\alpha }$ and $\bar{\beta }$. The Self-oscillation motion of the LCE film-mass system is controlled by these seven key parameters. This section investigates the effect of each key parameter on the onset condition, period and amplitude of the Self-sustained curling motion for LCE film-mass systems.

### 4.1. Effect of the Height of Electrolyte Solution

**Figure 5:**
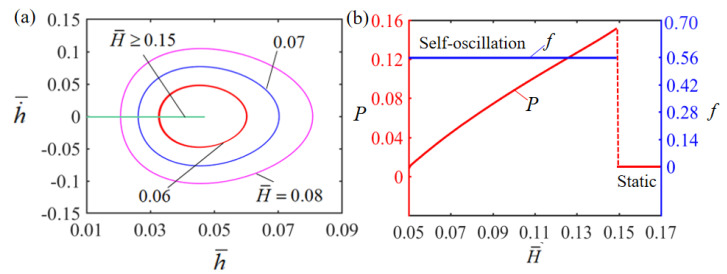
Effect of dimensionless height on the Self-sustained curling LCE film-mass system, with $\bar{g}=1.2$, $\bar{E}=2.5$, $\bar{A}=0.40$, $\bar{\alpha }=0.35$, $\bar{\dot{h}}=0$, $\bar{\beta }=0.001$ and ${\bar{T}}_{0}=0.1$. (**a**) Limit cycles; (**b**) amplitude and frequency.

Figure 5 illustrates how the dimensionless height $\bar{H}$ of the electrolyte solution affects Self-oscillation. In the numerical calculation, we set $\bar{g}=1.2$, $\bar{E}=2.5$, $\bar{A}=0.40$, $\bar{\alpha }=0.35$, $\bar{\dot{h}}=0$, $\bar{\beta }=0.001$ and ${\bar{T}}_{0}=0.1$. The limit cycles of the Self-sustained curling LCE film are plotted in Figure 5a for different heights, where a critical height of about 0.15 exists for the phase transition between the stationary and Self-oscillation patterns. When the initial height exceeds the critical height, there is not enough energy input to compensate for the damping dissipation and the system will eventually stay in a static equilibrium position and remain stationary. For $\bar{H}=0.06$, $\bar{H}=0.07$ and $\bar{H}=0.08$, the system can perform a Self-oscillation pattern. The effect of the dimensionless height $\bar{H}$ on the amplitude and frequency of Self-oscillation is also presented in Figure 5b. It can be observed that with the increase of dimensionless height, the frequency remains constant, while its amplitude gradually increases. This is attributed to the fact that the higher the solution height, the longer the resistance wire in the LCE film is continuously energized during Self-sustained curling, the more heat energy is generated, the more heat energy is input, and the greater the amplitude of Self-sustained curling. A critical height exists because there is a limit temperature difference in the electrothermally responsive LCE film; thus the LCE film eventually becomes stationary after the initial height exceeds the critical height. The solution height has no qualitative influence on the results, only a quantitative one.

### 4.2. Effect of the Gravitational Acceleration

**Figure 6:**
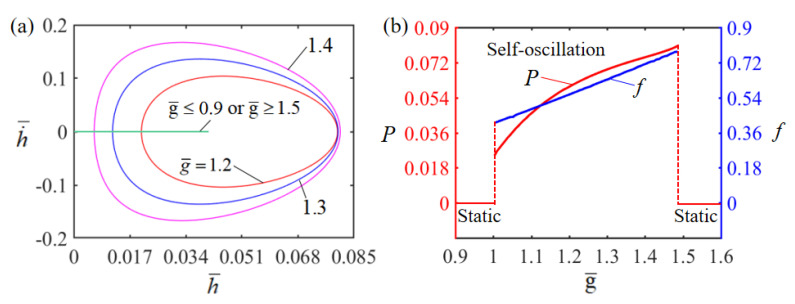
Effect of dimensionless gravitational acceleration on the Self-sustained curling LCE film-mass system with $\bar{H}=0.08$, $\bar{E}=2.5$, $\bar{A}=0.40$, $\bar{\alpha }=0.35$, $\bar{\dot{h}}=0$, $\bar{\beta }=0.001$ and ${\bar{T}}_{0}=0.1$. (**a**) Limit cycles; (**b**) amplitude and frequency.

Figure 6 illustrates the effect of dimensionless gravitational acceleration $\bar{g}$ on Self-oscillation. In the numerical calculation, we set $\bar{H}=0.08$, $\bar{E}=2.5$, $\bar{A}=0.40$, $\bar{\alpha }=0.35$, $\bar{\dot{h}}=0$, $\bar{\beta }=0.001$ and ${\bar{T}}_{0}=0.1$. The limit cycles of the Self-oscillation are plotted in Figure 6a for different gravitational accelerations $\bar{g}=1.2$, $\bar{g}=1.3$ and $\bar{g}=1.4$. For $0.8\leq \bar{g}\leq 1.5$, the system exhibits a Self-oscillation pattern. This result can be understood by the energy compensation between the net input energy and the damped dissipation. For small $\bar{g}$, the heat exchange is so fast that the LCE film deforms rapidly as it passes through the surface of the electrolyte solution. For large $\bar{g}$, the heat exchange is too slow and the LCE film barely deforms during Self-oscillation. As a result, the net work done by the electrical heat on the LCE film is too small to compensate the energy dissipated by the system damping to maintain Self-oscillation, and without sufficient energy input to compensate for damping dissipation, the LCE film will eventually stay in a static equilibrium position and maintain a stationary pattern. Figure 6b describes how the dimensionless gravitational acceleration $\bar{g}$ affects the amplitude and frequency of Self-oscillation. It is obvious that as the dimensionless gravitational acceleration increases, the frequency increases, which is consistent with physical intuition. Similarly, the Self-oscillation amplitude increases with increasing gravitational acceleration $\bar{g}$. Because of the factorless gravitational acceleration $\bar{g}=\frac{g\tau }{L}$, the length of the LCE film can be varied to meet the requirements of practical applications.

### 4.3. Effect of the Elasticity Modulus

**Figure 7:**
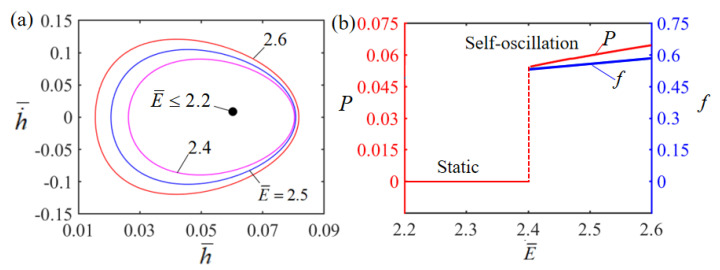
Effect of dimensionless elastic modulus on a Self-sustained curling LCE film-mass system with $\bar{H}=0.08$, $\bar{g}=1.2$, $\bar{A}=0.40$, $\bar{\alpha }=0.35$, $\bar{\dot{h}}=0$, $\bar{\beta }=0.001$ and ${\bar{T}}_{0}=0.1$. (**a**) Limit cycles; (**b**) amplitude and frequency.

The effect of dimensionless elastic modulus $\bar{E}$ on Self-oscillation is presented in Figure 7. In the numerical calculation, we set $\bar{H}=0.08$, $\bar{g}=1.2$, $\bar{A}=0.40$, $\bar{\alpha }=0.35$, $\bar{\dot{h}}=0$, $\bar{\beta }=0.001$ and ${\bar{T}}_{0}=0.1$ The limit cycles of the Self-oscillation are plotted in Figure 7a for different dimensionless elasticity moduli, where a critical elastic modulus of about 2.0 exists for the phase transition between the stationary and Self-oscillation patterns. When the dimensionless elasticity modulus is less than the critical value, the LCE film eventually stays in a static equilibrium position and remains stationary. This is because for small E, the LCE film is very soft, resulting in little contact force and little net work carried out by the contact force during contact; therefore, the energy input cannot compensate for the damping dissipation to maintain Self-oscillation. For $\bar{E}=2.4$, $\bar{E}=2.5$ and $\bar{E}=2.6$, the LCE film presents a Self-oscillation pattern. The dependence of elastic modulus on Self-oscillation amplitude and frequency is presented in Figure 7b. It is clear that as the dimensionless elastic modulus increases, both the frequency and the amplitude gradually increase. This is due to the fact that a larger dimensionless elastic modulus will provide a larger elastic force and a smaller intrinsic period, which is consistent with physical intuition. In fact, from Equation (9), it is generally predicted that the frequency follows the square-root law as described by the first-order cantilever beam theory, i.e., $f\propto \sqrt{E}$, which is consistent with the numerical results shown in Figure 7b. It is of interest to note that from an experimental point of view, the LCE material itself may become harder or softer as the temperature changes. Then, this will be intrinsically related to mechanical parameters such as the elastic modulus of the LCE material itself, and in turn affects the behaviors of Self-oscillation, which deserves to be further investigated in the future.

### 4.4. Effect of the Limit Temperature

**Figure 8:**
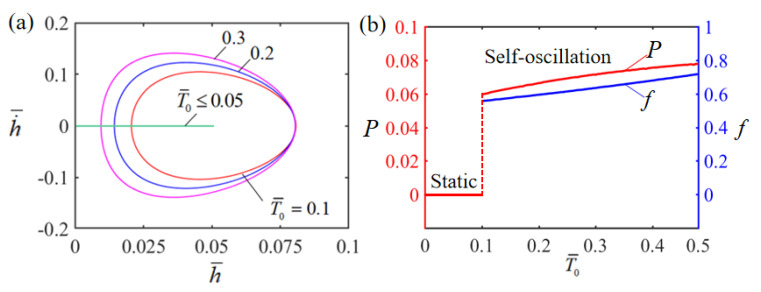
Effect of dimensionless limit temperature on the Self-sustained curling LCE film-mass system with $\bar{H}=0.08$, $\bar{g}=1.2$, $\bar{E}=2.5$, $\bar{A}=0.40$, $\bar{\alpha }=0.35$, $\bar{\dot{h}}=0$ and $\bar{\beta }=0.001$. (**a**) Limit cycles; (**b**) amplitude and frequency.

Figure 8 displays the effect of the dimensionless limit temperature ${\bar{T}}_{0}$ on Self-oscillation. In the numerical calculation, we set $\bar{H}=0.08$, $\bar{g}=1.2$, $\bar{E}=2.5$, $\bar{A}=0.40$, $\bar{\alpha }=0.35$, $\bar{\dot{h}}=0$ and $\bar{\beta }=0.001$. The limit cycles of Self-oscillation are plotted in Figure 8a for different limit temperatures. There is a presence of a critical limit temperature valued at about 0.05 for the phase transition between stationary and Self-oscillation patterns. When the dimensionless limit temperature is less than the critical value, there is not enough energy input to compensate for the damping dissipation and the LCE film will eventually stay in the static equilibrium position and maintain a stationary state. For ${\bar{T}}_{0}=0.1$, ${\bar{T}}_{0}=0.2$ and ${\bar{T}}_{0}=0.3$, the LCE film will perform a Self-oscillation motion. The effect of the dimensionless limit temperature ${\bar{T}}_{0}$ on Self-oscillation amplitude and frequency is depicted in Figure 8b. It can be observed that the amplitude and frequency increase as the dimensionless limit temperature ${\bar{T}}_{0}$ increases. This is owing to the fact that the higher the limit temperature generated by the current, the more heat energy is generated, and the higher the Self-oscillation amplitude of the LCE film. 

### 4.5. Effect of the Curvature Coefficient

**Figure 9:**
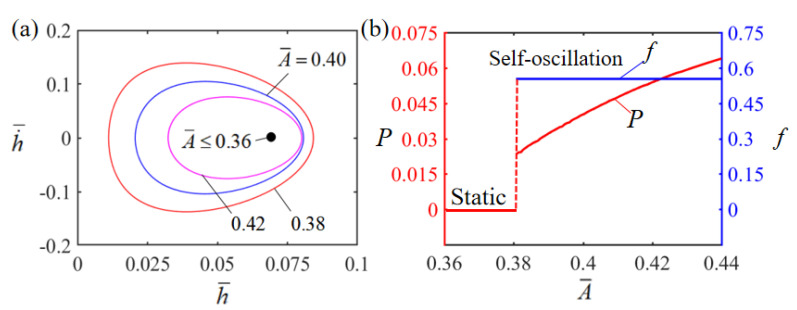
Effect of dimensionless curvature coefficient on the Self-sustained curling LCE film-mass system with $\bar{H}=0.08$, $\bar{g}=1.2$, $\bar{E}=2.5$, $\bar{\alpha }=0.35$, $\bar{\dot{h}}=0$, $\bar{\beta }=0.001$ and ${\bar{T}}_{0}=0.1$. (**a**) Limit cycles; (**b**) amplitude and frequency.

The effect of the dimensionless curvature coefficient $\bar{A}$ on Self-oscillation is illustrated in Figure 9. In the numerical calculation, we set $\bar{H}=0.08$, $\bar{g}=1.2$, $\bar{E}=2.5$, $\bar{\alpha }=0.35$, $\bar{\dot{h}}=0$, $\bar{\beta }=0.001$ and ${\bar{T}}_{0}=0.1$. Figure 9a plots the limit cycles of Self-oscillation for different dimensionless curvature coefficients, where a critical value of about 0.36 for the curvature coefficient exists to trigger a Self-oscillation pattern. When the dimensionless curvature coefficient is less than the critical value, the system does not have enough energy input to compensate for the damping dissipation, and it will eventually stay in the static equilibrium position and maintain a stationary pattern. For $\bar{A}=0.38$, $\bar{A}=0.40$ and $\bar{A}=0.42$, a Self-oscillation pattern is triggered. The effect of the dimensionless curvature coefficient $\bar{A}$ on the Self-oscillation amplitude and frequency is presented in Figure 9b. The amplitude is observed to increase with the increase in the dimensionless curvature coefficient $\bar{A}$. This is due to the fact that the greater the curvature coefficient, the greater the work done by the LCE film on the mass block. However, as the dimensionless thermal curvature coefficient increases, the frequency exhibits no significant change. This is because the dimensionless thermal curvature coefficient only reflects system deformation without changing the intrinsic frequency of the system.

### 4.6. Effect of the Thermal Shrinkage Coefficient

**Figure 10:**
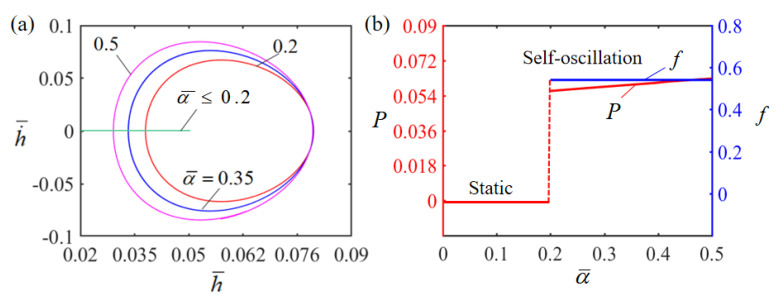
Effect of dimensionless thermal shrinkage coefficient on the Self-sustained curling LCE film-mass system with $\bar{H}=0.08$, $\bar{g}=1.2$, $\bar{E}=2.5$, $\bar{A}=0.40$, $\bar{\dot{h}}=0$, $\bar{\beta }=0.001$ and ${\bar{T}}_{0}=0.1$. (**a**) Limit cycles; (**b**) amplitude and frequency.

Figure 10 describes the effect of the dimensionless thermal shrinkage coefficient $\bar{\alpha }$ on Self-oscillation. In the numerical calculation, we set $\bar{H}=0.08$, $\bar{g}=1.2$, $\bar{E}=2.5$, $\bar{A}=0.40$, $\bar{\dot{h}}=0$, $\bar{\beta }=0.001$ and ${\bar{T}}_{0}=0.1$. The limit cycles of the Self-oscillation are plotted in Figure 10a for different dimensionless thermal shrinkage coefficients. There exists a critical thermal shrinkage coefficient of about 0.1 for triggering Self-oscillation. When the dimensionless thermal shrinkage coefficient is less than the critical value, there is not enough energy input to compensate for the damping dissipation and the LCE film will eventually stay in the static equilibrium position, presenting a stationary pattern. For $\bar{\alpha }=0.2$, $\bar{\alpha }=0.35$ and $\bar{\alpha }=0.5$, the LCE film can perform a Self-oscillation pattern. The effect of the dimensionless thermal shrinkage coefficient $\bar{\alpha }$ on Self-oscillation amplitude and frequency is displayed in Figure 10b. It can be clearly seen that the amplitude increases with the increase in thermal shrinkage coefficient $\bar{\alpha }$. This is attributed to the fact that the larger the thermal shrinkage coefficient $\bar{\alpha }$, the greater the work done by the LCE film on the mass block. As the thermal shrinkage coefficient increases, the Self-oscillation frequency does not change much, because the thermal shrinkage coefficient only reflects the system deformation without changing the intrinsic frequency of the system.

### 4.7. Effect of the Damping Factor

**Figure 11:**
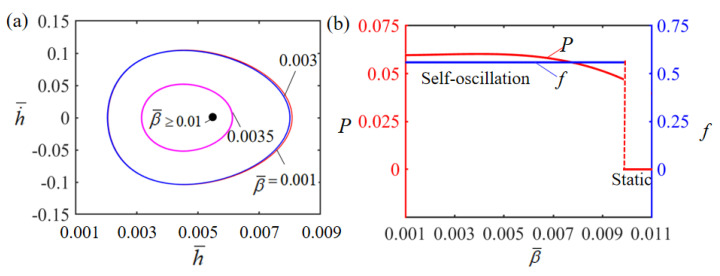
Effect of dimensionless damping factor on the Self-sustained curling LCE film-mass system with $\bar{H}=0.08$, $\bar{g}=1.2$, $\bar{E}=2.5$, $\bar{A}=0.40$, $\bar{\alpha }=0.35$, $\bar{\dot{h}}=0$ and ${\bar{T}}_{0}=0.1$. (**a**) Limit cycles; (**b**) amplitude and frequency.

Figure 11 illustrates how the dimensionless damping factor $\bar{\beta }$ influences Self-oscillation. In the numerical calculation, we set $\bar{H}=0.08$, $\bar{g}=1.2$, $\bar{E}=2.5$, $\bar{A}=0.40$, $\bar{\alpha }=0.35$, $\bar{\dot{h}}=0$, and ${\bar{T}}_{0}=0.1$. The limit cycles of the Self-oscillation are plotted in Figure 11a for different damping factors, where a critical damping coefficient of about 0.01 exists for the phase transition between stationary and Self-oscillation patterns. When the damping coefficient is larger than the critical value, the damping dissipation of the system is too large and exceeds the mechanical energy converted from the input thermal energy, and the LCE film will eventually stay in the static equilibrium position and remain stationary. However, for $\bar{\beta }=0.001$, $\bar{\beta }=0.003$ and $\bar{\beta }=0.0035$, a Self-oscillation pattern can be triggered. Figure 11b displays the effect of the dimensionless damping factor $\bar{\beta }$ on the Self-oscillation amplitude and period. It is obvious that the amplitude decreases with the increase in damping factor $\bar{\beta }$. This is owing to the fact that as the damping factor $\bar{\beta }$ increases, the more energy is dissipated by the system due to the damping effect. Meanwhile, the damping coefficient has little influence on the intrinsic frequency of the system, and the Self-oscillation frequency presents no significant variation as the damping factor increases.

In this section, the effects of several key dimensionless parameters on the amplitude and frequency of the Self-excited oscillation of the system have been investigated, and are summarized in Table 3. These results can provide guidance for designing Self-oscillating systems and modulating the behaviors of Self-oscillation in applications.

## 5. Conclusions

Self-excited oscillation systems can generate periodic motion in response to a constant external stimulus and have potential applications in areas such as micro devices, autonomous robots, sensors and energy-generating devices. In this paper, we propose a Self-sustained curling LCE film-mass system based on a conventional electrothermally driven shrinkage material, which can achieve Self-oscillation in steady-state circuits. Based on the existing LCE dynamics model and the dynamic boundary problem of contact surfaces, a nonlinear dynamics model for a Self-sustained curling LCE film-mass system in a steady-state circuit is developed. The fourth-order Runge–Kutta method is applied for a numerical solution, and the calculation results show that there are two motion patterns, namely the stationary pattern and Self-oscillation pattern. Self-sustained curling of the LCE film-mass system can be triggered by controlling several key system parameters, including electrolyte solution height, gravitational acceleration, equivalent elastic modulus, limit temperature, curvature coefficient, thermal shrinkage coefficient and damping factor, with the Self-oscillation period and amplitude also being controlled by these parameters. These results of this paper are expected to be validated in future experimental works, and may provide a new thinking approach for the design of Self-excited oscillation systems, which will not only deepen the understanding of Self-excited oscillations, but also have broad application prospects in energy harvesting, power generation, monitoring, soft robotics, medical devices, and micro and nano devices.

## Figures and Tables

**Table 1:** Material properties and geometric parameters.

Parameter	Definition	Value	Unit
α	Thermal shrinkage coefficient of the LCE material	0–0.5	/
β	Damping factor	0.001~0.01	kg/s
q	Heat generated by current	0–~10	J /s
$I_{Z}$	Principal moment of inertia	10^−7^	$m^{4}$
$\rho_{c}$	Specific heat capacity of the LCE material	1000~4500	$J\;/\left(\mathrm{kg}℃\right)$
a	Thickness of the LCE film	10^−4^	m
τ	Characteristic time of heat exchange between the LCE film and the environment	0.001~0.1	s
$T_{0}$	Limit temperature difference of the LCE film	0–20	℃
H	Height of the electrolyte solution	0~0.001	m
$\varepsilon_{0}$	Length of the LCE film	0.01~0.02	m
g	Gravitational acceleration	10	${m/s}^{2}$
E	The total elastic modulus of the LCE layer and the insulation layer	1–10	Mpa
m	Mass of the mass block	0.001	kg

**Table 2:** Dimensionless parameters.

Parameter	$\bar{H}$	$\bar{g}$	$\bar{E}$	${\bar{T}}_{0}$	$\bar{A}$	$\bar{\alpha }$	$\bar{\beta }$
Value	0.05~0.1	1.1~1.3	2.4~2.6	0.1~0.5	0.38~0.45	0.2~0.5	0.01~0.1

**Table 3:** Effects of several key dimensionless parameters.

Dimensionless Parameter	Amplitude	Frequency
$\bar{H}$	increases with increasing $\bar{H}$	not affected by $\bar{H}$
$\bar{g}$	increases with increasing $\bar{g}$	increases with increasing $\bar{g}$
$\bar{E}$	increases with increasing $\bar{E}$	increases with increasing $\bar{E}$
${\bar{T}}_{0}$	increases with increasing ${\bar{T}}_{0}$	increases with increasing ${\bar{T}}_{0}$
$\bar{A}$	increases with increasing $\bar{A}$	not affected by $\bar{A}$
$\bar{\alpha }$	increases with increasing $\bar{\alpha }$	not affected by $\bar{\alpha }$
$\bar{\beta }$	decreases with increasing $\bar{\beta }$	not affected by $\bar{\beta }$

## Data Availability

The data that support the findings of this study are available upon reasonable request from the authors.
